# Prolonged Subdural Infusion of Kynurenic Acid Is Associated with Dose-Dependent Myelin Damage in the Rat Spinal Cord

**DOI:** 10.1371/journal.pone.0142598

**Published:** 2015-11-12

**Authors:** Wojciech Dabrowski, Jacek M. Kwiecien, Radoslaw Rola, Michal Klapec, Greg J. Stanisz, Edyta Kotlinska-Hasiec, Wendy Oakden, Rafal Janik, Margaret Coote, Benicio N. Frey, Waldemar A. Turski

**Affiliations:** 1 Department of Anaesthesiology and Intensive Therapy Medical University, Lublin, Poland; 2 Department of Pathology and Molecular Medicine, M. deGroote School of Medicine, McMaster University, Hamilton, Ontario, Canada; 3 Department of Neurosurgery and Paediatric Neurosurgery Medical University, Lublin, Poland; 4 Department of Orthopaedic and Traumatology Medical University, Lublin, Poland; 5 Department of Medical Biophysics, University of Toronto, Ontario, Canada; 6 Physical Sciences Platform, Sunnybrook Research Institute, Ontario, Canada; 7 Department of Psychiatry and Behavioural Neurosciences, M. deGroote School of Medicine, McMaster University, Hamilton, Ontario, Canada; 8 Department of Experimental and Clinical Pharmacology, Medical University, Lublin, Poland; Washington University, UNITED STATES

## Abstract

**Background:**

Kynurenic acid (KYNA) is the end stage metabolite of tryptophan produced mainly by astrocytes in the central nervous system (CNS). It has neuroprotective activities but can be elevated in the neuropsychiatric disorders. Toxic effects of KYNA in the CNS are unknown. The aim of this study was to assess the effect of the subdural KYNA infusion on the spinal cord in adult rats.

**Methods:**

A total of 42 healthy adult rats were randomly assigned into six groups and were infused for 7 days with PBS (control) or 0.0002 pmol/min, 0.01 nmol/min, 0.1 nmol/min, 1 nmol/min, and 10 nmol/min of KYNA per 7 days. The effect of KYNA on spinal cord was determined using histological and electron microscopy examination. Myelin oligodendrocyte glycoprotein (MOG) was measured in the blood serum to assess a degree of myelin damage.

**Result:**

In all rats continuous long-lasting subdural KYNA infusion was associated with myelin damage and myelin loss that was increasingly widespread in a dose-depended fashion in peripheral, sub-pial areas. Damage to myelin sheaths was uniquely related to the separation of lamellae at the intraperiod line. The damaged myelin sheaths and areas with complete loss of myelin were associated with limited loss of scattered axons while vast majority of axons in affected areas were morphologically intact. The myelin loss-causing effect of KYNA occurred with no necrosis of oligodendrocytes, with locally severe astrogliosis and no cellular inflammatory response. Additionally, subdural KYNA infusion increased blood MOG concentration. Moreover, the rats infused with the highest doses of KYNA (1 and 10 nmol/min) demonstrated adverse neurological signs including weakness and quadriplegia.

**Conclusions:**

We suggest, that subdural infusion of high dose of KYNA can be used as an experimental tool for the study of mechanisms of myelin damage and regeneration. On the other hand, the administration of low, physiologically relevant doses of KYNA may help to discover the role of KYNA in control of physiological myelination process.

## Introduction

Kynurenic acid (KYNA) is an endogenous, neuroactive, end stage product of tryptophan metabolism, which in the central nervous system (CNS) is mainly synthesized and liberated by astrocytes [1,2]. KYNA acts as a wide-spectrum endogenous antagonist of N-methyl-D-aspartate (NMDA) and of α7 nicotinic acetylcholine (α7nACh) receptors [3,4]. Physiological concentration of the human cerebrospinal fluid (CSF) KYNA ranges between 1–5 nM in human, 6 nM in monkey and 32 nM in adult gerbil [5–9]. The concentration of KYNA in the rat CSF has not been determined, however its concentration in brain has ranged between 0.5–1 nM [5]. Elevated level of KYNA in CSF have been reported in several neurological and psychiatric disorders, such as depression, Huntington’s disease, bipolar disorders, Alzheimer’s disease, Parkinson’s disease, epilepsy and schizophrenia [7,9–13]. At micromolar concentrations KYNA exerts a neuroinhibitory effect, while at nanomolar concentration KYNA acts as a facilitator in the rat hippocampus [9].

Several studies have documented a neuroprotective effect of KYNA or its analogues after CNS injury [14–17]. In an experimental model of spinal cord injury, subdural infusion of glucosamine-KYNA was shown to improve locomotor recovery and prevented secondary destruction of spinal cord [16]. This analogue of KYNA was also shown to significantly reduce the average length of the post-traumatic lesion. Such effect of glucosamine-KYNA infusion was more pronounced in the gray matter than in the white matter [16]. Notably, in all of the above-mentioned studies the neuroprotective effects of a single dose of KYNA were analyzed after the CNS injury. However, the effects of prolonged administration of KYNA on a healthy CNS remain largely unknown. There is substantial amount of data showing increased levels of KYNA in neuropsychiatric disorders in humans, as well as in animals models of chronic neurodegenerative diseases [17], which suggests that elevated KYNA may be associated with neuropsychiatric conditions. Prolonged increase in KYNA concentration results in an impaired memory [18]. Noteworthy, disorders in memory frequently correspond with myelin injury [19]. An increase in brain KYNA level has led to significant neurochemical and morphological disorders affecting different cognitive dysfunctions [20]. An intrathecal KYNA administration has resulted in a motor dysfunction and antinociception [21,22]. Moreover, a continuous intrathecal infusion of KYNA at the doses 0.1–4 μg/min for 60 min has resulted in motor paralysis, and this effect was temporary and reversible [23]. The intracerebroventricular administration of KYNA reduces spontaneous EMG activity in dose-dependent manner and causes muscle relaxation in genetically spastic rats [24]. However, all these studies presents a behavioural effect of KYNA administration, and the histological effects of KYNA on myelin sheaths and on oligodendrocytes remain unknown. Recently, Lisak et al., (2015) described death of neurons incubated for 24 h in medium containing KYNA [25]. Based on these observations we assume that an increase in KYNA concentration in CSF may cause damage in central nervous system, however, a toxic dose of KYNA has been not precised. Since the elimination rate of KYNA from the brain is rapid [26], the aim of the present study was to assess the effects of continuous long-lasting subdural infusion of KYNA on the spinal cord in healthy rats with histological and electron microscopic analyse, and determine its effect on the serum levels of myelin oligodendrocyte glycoprotein (MOG).

## Material and Methods

The animal experiments were approved by the Animal Research Ethics Board at McMaster University, Ontario, Canada according to the Canadian Council of Animal Care guidelines. A total of 42 healthy adult Long Evans rats from both sexes, 4–7 months old, weighing 250 – 400g, were used in these experiments. The rats were randomly assigned into six groups (n = 7). The rats were housed individually in a pathogen-free facility and were offered rat chow and tap water *ad libitum*.

### Subdural Infusion

**Fig 1:**
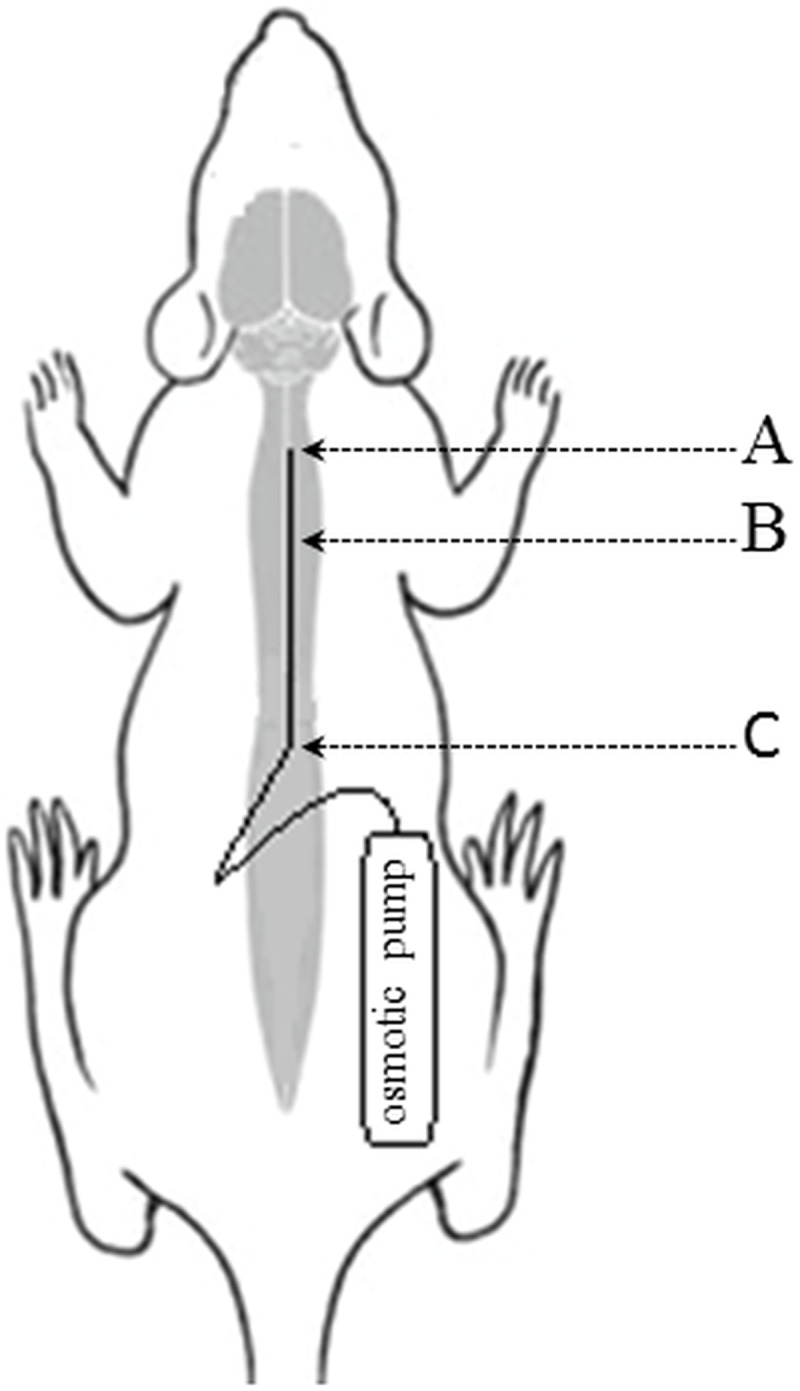
The schematic presentation of intrathecal catheter placement. A–C7 –Th1 level of spinal cord, B–intrathecal catheter, C–a place of surgery.

The rats were induced and maintained with isoflurane admixed at 3% to oxygen. The animals breathed spontaneously. The skin over the dorsum was shaved and disinfected and laminectomy performed over the lumbar region (L_2_) of the spine and the exposed dura was cut with a 25 ga needle. A rat intrathecal catheter (Alzet, Durect Corporation, Cupertino, CA) 6.5 cm long, was carefully inserted into the subdural space over the dorsal spinal cord to approximate C7-Th1 level of spinal cord (Fig 1). The catheter was fixed in place by suturing it to the adjacent lumbar muscles. After the steel guide was removed, the catheter was connected with an osmotic pump with the infusion time of 1 week and volume 2 mL (Alzet) that was pre-loaded with KYNA (Sigma Aldrich) solution or phosphate buffered saline (PBS)–control. 0.473 g of KYNA was diluted in 50 mL of PBS and slowly titrated with 1 normal NaOH to raise the pH to 7.5. Diluted KYNA was filter-sterilized with 0.2 micron syringe-top miliQ filter before injected into the osmotic pumps and next 7 osmotic pumps were preloaded with 2 mL of KYNA solution (50 mM in each pump). This dilution was considered a stock for the lower dilutions and it was prepared immediately before the loading of the pumps. The serial dilutions were prepared by diluting the stock solutions with PBS to obtain 1:10 dilution for 5 mM KYNA, 1:100 dilution for 0.5 mM KYNA, 1:1,000 dilution for 0.05 mM KYNA. To obtain 1 nM concentration of KYNA a series of stepwise dilutions was prepared using 0.05 mM KYNA as a stock solution.

The osmotic pump was placed subcutaneously on the flank of the rat. The spinal muscles were apposed with absorbable sutures over the laminectomy and the skin was closed with stainless steel staples. After the surgery all rats received subcutaneous ketoprofen (Anafen, Merial Canada, Inc., Baie d’Urfe, Quebec, CA) at the dose of 0.2–0.3 mL for postoperative analgesia and a subcutaneous injection of 5 mL saline. The injections of ketoprofen were repeated once daily for 2 more days. Rats with postoperative haematuria and urinary bladder distension were treated using 2.5% enrofloxacine (Baytril, Bayer HealthCare, CA) at the dose 5 mg/kg body w.t..

Immediately after the catheter insertion and implantation of the osmotic pump, rats received subdural infusion of KYNA or PBS for seven days except for rats dosed with the highest concentration of KYNA that were terminated in poor health at 5 days after the onset of infusion. Rats were infused with 0.0002 pmol/min, 0.01 nmol/min, 0.1 nmol/min, 1 nmol/min and 10 nmol/min of KYNA per 7 days. Rats infused with 10 nmol/min of KYNA were maintained until the endpoint: general weakness, quadriplegia, dehydration, anorexia, hypothermia, that was reached at the day 5. All rats were examined twice a day and assessed for weakness and decline in reaction to the toe pinch in the hind limbs.

For the whole body perfusion, the rats were deeply anaesthetized with the intraperitoneal injection of 100 mg/kg body weight sodium barbital (Ceva, France) and the chest opened. A volume of 4–5 mL of blood was collected from the left ventricle for the myelin protein assay in the serum. A dose of 100 IU heparine sodium was injected into the left ventricle and the cannula inserted into the left ventricle while the right auricle was cut open to allow for wash-out of blood with lactated Ringer’s solution followed by a Karnowski’s fixative for histology and electron microscopy [27].

### Serum Myelin Oligodendrocyte Glycoprotein (MOG)

Blood from the left cardiac ventricle was collected into disposable plastic syringes and aliquoted into 1.5 mL Eppendorf microtubes for 45 min. The serum was separated by a 15 min spin at 3,000G and carefully removed from above the cells prior to storing at -70C until assayed.

The rat serum Myelin Oligodendrocyte Glycoprotein (MOG) was analyzed with a double-sandwich ELISA technique (MyBioSource Inc., San Diego, CA). The experiments were performed in duplicate by an experienced lab technician who was blinded to the experimental design. The ELISA plate was pre-coated with the appropriate monoclonal antibody and the rat serum samples and the kit calibrators were added to the plate wells and incubated according to the manufacture’s protocol, to allow any antigen present to bind to the pre-coated antibody wells. Subsequently, the plate was washed with PBS to remove any unbound antigen and a detection biotin labelling antibody was added to the coated plate wells to bind to the remaining antigen, incubated and washed out with PBS. An enzyme-linked secondary antibody was then added to bind to the detecting antibody, incubated and washed out to remove the unbound antibody-enzyme conjugates. TMB substrate was then added and incubated for 30 minutes to be converted by the enzyme into a colour signal, and the reaction was stopped with the addition of sulphuric acid. The plates were read in a Microplate reader (Thermo–Scientific, 450 detection wavelength filter) within 10 minutes. The software program Multiskan Ascent was used to compute the results plotting standard curves with the known calibrators concentrations versus the optical density values measured. The serum MOG concentrations were then interpolated from the standard curves. The MOG assay detection range was 31.25 pg/ml—2000 pg/ml, the sensitivity was 7.81 pg/ml, the intra and inter variation were 8% and 10%, respectively.

### Histology and Electron Microscopy

The tissues of the brain, optic nerve and the spinal cord were removed carefully and post-fixed in Karnowski’s [27]. A cross section of the cervical spinal cord 9 mm caudal to the cerebellum was collected and used for morphological analyses. For histology, 1 μm thick epon-embeded sections were cut with a glass knife, mounted on a glass slide and stained with toluidine blue. These sections were analyzed under a Nikon Eclipse 50i microscope. Silver gray ultrathin sections from Epon-embedded portions of the spinal cord were mounted on Formvar coated copper grids, stained with uranyl acetate and lead citrate and examined under a Jeol 1200EX Biosystem transmission electron microscope.

### Statistics

The data were analyzed using Statistics 9.0.0 software (IBM, Chicago, USA). Initially, normal distribution of the serum MOG concentrations were analyzed by the Shapiro-Wilk test. Means and standard deviations (SD) were calculated for normally distributed data and Student unpaired *t*-test was used to compare the variables. ANOVA unvariate analysis with post hoc Dunnett’s test was used for analysis of the differences between studied groups.

## Results

All rats recovered well from the surgery. The rats infused with 0.0002 pmol/min and 0.01 nmol/min of KYNA had no abnormal clinical signs during the one week of infusion. They slightly lost body weight however they ate and drank water normally. The urinary bladder was not distended in all rats and there was no blood in urine. The rats infused with 0.1 nmol/min of KYNA had mild to moderate hind end weakness from day 5–7. They moderately lost body weight, were anorexic, had reduced drinking, were moderately imbalanced, had distended urinary bladder. Moderate haematuria was noted in two rats and severe haematuria was noted in one. The rats infused with 10 nmol/min of KYNA did progressively poorly and had progressively severe generalized weakness from the day 3 which was combined with lethargy and hypothermia. These rats developed complete hind end paralysis at day 5 post-op. All of them were anorexic and dehydrated. The urinary bladder was distended and severe haematuria was noted in all 5 rats.

### Neuropathology

**Fig 2:**
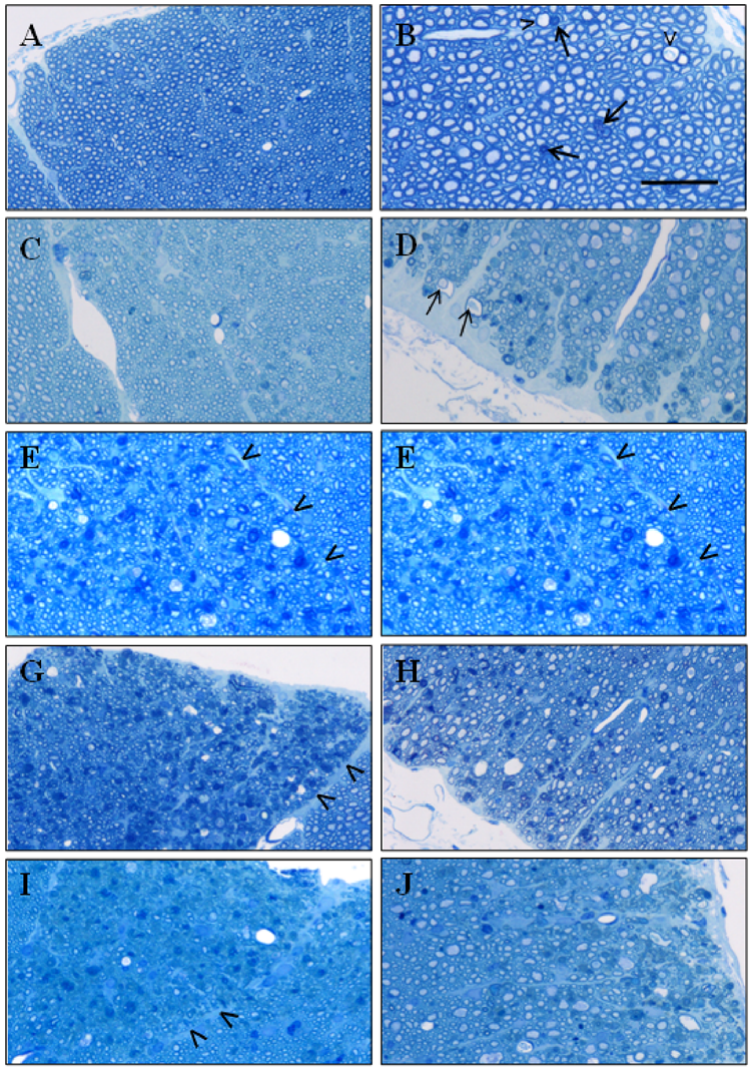
Histomicrographs of the sections of the cervical spinal cord of rats infused intrathecally with kynurenic acid (KYNA). Toluidine blue (TB) stain. In the dorsal–A, and lateral–B, columns from a rat treated with 0.0002 pmol/min of KYNA per 7 days, there are sparse, individual axons with thickened myelin sheaths staining dark with TB. In the dorsal column–C of a rat infused with 0.01 nmol/min of KYNA per 7 days there are scattered myelin sheaths that are thickened, whereas in the lateral column–D, in the sub-pial areas, there are greater numbers of thickened myelin sheaths and there are also large axons that have dilated and attenuated myelin sheaths (arrows). The numbers of abnormal myelin sheaths appear to increase in the fasciculus gracilis (delineated by the arrow heads) and in the subpial areas of the dorsal and of the lateral and ventral columns in rats infused with; 0.1 nmol/min of KYNA per 7 days, E–dorsal column, F–lateral column; 1 nmol/min of KYNA per 7 days, G–dorsal column, H–ventral column; and 10 nmol/min of KYNA infused per 5 days, I–dorsal column, J–lateral column. Size bars– 50 μM.

In the subpial areas of cervical spinal cord of rats treated with KYNA (0.0002 pmol/min– 10 nmol/min), there were scattered axons with myelin sheaths that had increased staining with toluidine blue. The numbers of abnormal myelin sheaths increased with the elevation of the dose of KYNA. The increase of the numbers of abnormal myelin sheaths correlated with the severity of locally diffuse astrogliosis and the thickness of the glia limitans. In the dorsal column there was a sharp demarcation between the fasciculus gracilis that had abnormal myelin sheaths from the fasciculus cuneatus that did not (Fig 2).

**Fig 3:**
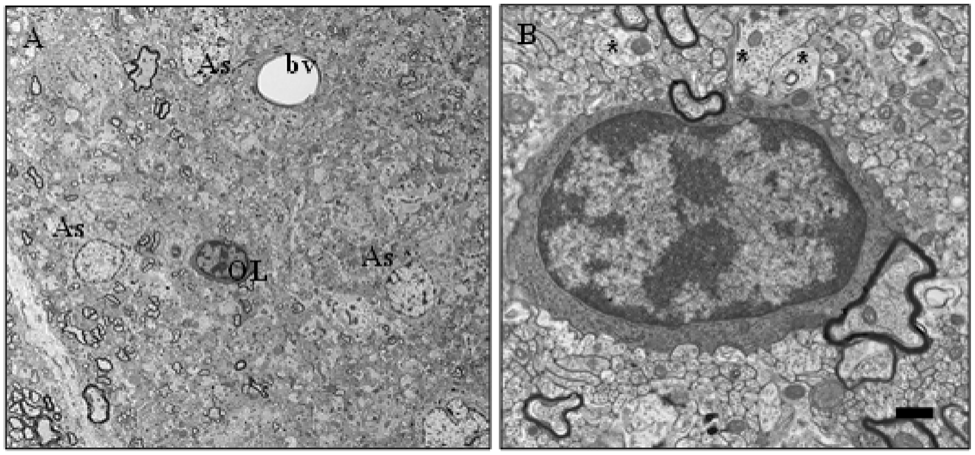
Electron micrographs of severe demyelination in the area of the fasciculus gracilis of the dorsal column in the rat treated with the intrathecal infusion of 10 nmol/min of kynurenic acid (KYNA) per 5 days. A–in the area of severe demyelination, most of axons are naked, there are 3 astrocytes (As), one oligodendrocyte (OL) and a small blood vessel (bv). B–on higher magnification, the oligodendrocyte appears to have a compact cytoplasm devoid of processes; it is surrounded by many naked axons, some of the diameter greater than 2 μM (asterices) and a few myelinated axons. Size bars; A– 10 μM, B– 2 μM.

Transmission electron microscopy revealed that in the fasciculus gracilis of the rats treated with the 10 nmol/min of KYNA there was a widespread complete loss of myelin with preservation of normal morphology of axons (Fig 3). There were scattered individual and small clusters of thin myelin sheaths in the area of myelin loss. Oligodendrocytes had retracted processes and small amount of cytoplasm poor in organelles. The astrocytes were hypertrophied.

**Fig 4:**
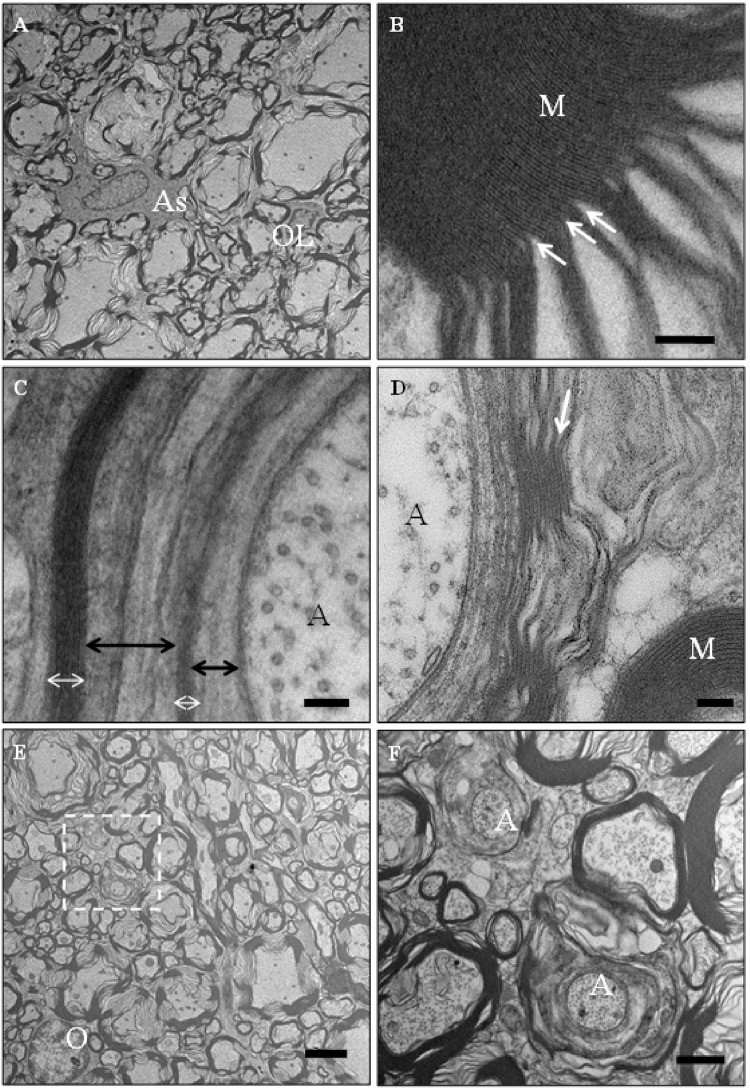
Electron micrographs of damaged myelin sheaths from the spinal cord of rats infused intrathecally with kynurenic acid (KYNA) for 7 days. A–an area from the dorsal column of a rat infused with 0.01 nmol/min of KYNA with an astrocyte (As) and an oligodendrocyte (OL) surrounded by damaged myelin sheaths. B–in this detail of A delineated by the white box, a segment of well compacted thick myelin sheath (Ms) passes into a segment were all lamellae are widely separated due to disintegration of compaction at the intraperiod line indicated by arrows. C–an example of a damaged myelin sheath from a single axon (Ax) from the lateral column of the rat infused with 10 nmol/min KYNA were a few well compacted lamellae (white double headed arrows) are widely separated by uncompacted lamellae (black arrows). D–in the lateral column of a rat infused with 1 nmol/min of KYNA, an axon (Ax) has a damaged myelin with segmental loss of compaction due to separation of lamellae at the intraperiod line (white arrow). There is a well-compacted thick myelin sheath (Ms) in the adjacent axon. E–lateral column from a rat infused with 0.0002 pmol/min of KYNA per 7 days, with multiple myelin sheaths showing the segmental loss of compaction and one oligodendrocyte (OL). The box indicates the area displayed in higher magnification in F–with two axons (Ax) surrounded by uncompacted myelin lamellae. Size bars; A, E– 5 μM, B, C, D– 100 nM, F– 1 μM.

In the subpial areas of the dorsal column in rats treated with 0.01 nmol/min– 1 nmol/min of KYNA and in the lateral and ventral columns of the rats treated with 0.01 nmol/min– 10 nmol/min of KYNA there were axons with degenerating changes in the myelin sheath whose proportion increased with the increase in the concentration of KYNA (Fig 4). In cross sections the myelin sheaths appeared swollen, with the lamellae dissociated in a segmental or diffuse fashion individually or in variably thick stacks (Fig 4C and 4D). The separation of the myelin lamellae was consistently at the intraperiod line of the sheath (Fig 4B). Although most of the axons with the myelin sheath appeared morphologically unchanged, there were rare scattered axons with degenerative changes of the swollen sheath and with atrophied, dark, abnormal axon (not shown). There were scattered oligodendrocytes with retracted processes and small amount of cytoplasm. There was locally diffuse astrogliosis. In the subpial areas of the lateral and ventral columns of rats treated with 0.0002 pmol/min of KYNA there were rare scattered swollen sheaths with the characteristic separation of the lamellae at the intraperiod line similar to that described above.

### Serum Myelin Oligodendrocyte Glycoprotein (MOG)

**Fig 5:**
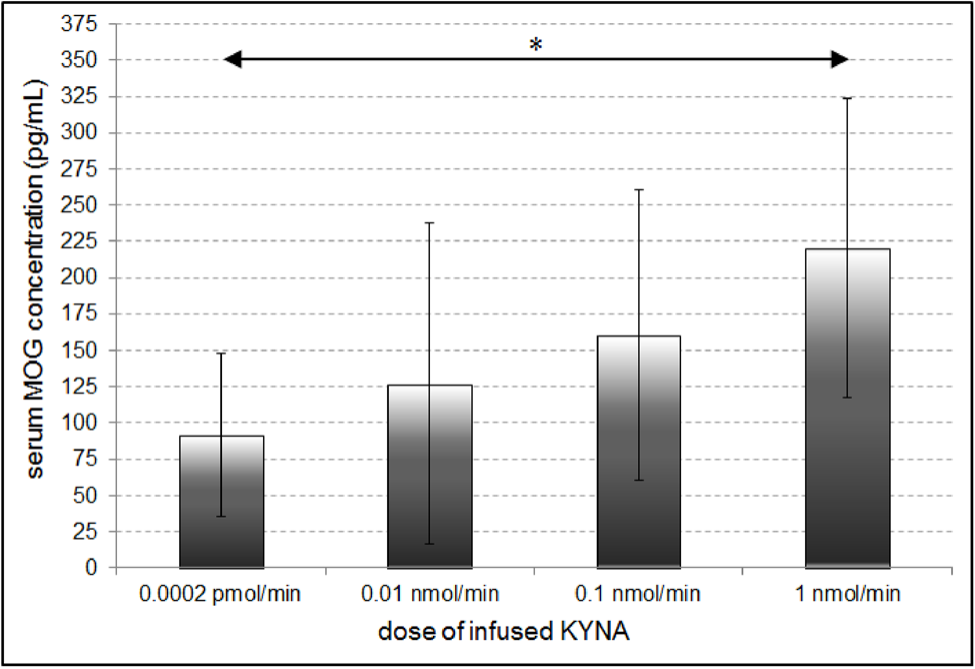
Changes in serum myelin oligodendrocyte glycoprotein (MOG) concentration in rats received intrathecal infusion of saline and kynurenic acid (KYNA) at the doses: 0.0002 pmol/min, 0.01 nmol/min, 0.1 nmol/min, 1 nmol/min per 7 days. * p<0.05 –significant difference in serum MOG concentration in rats received 1 nmol/min of KYNA in comparison with saline.

One-way analysis of variance showed that serum MOG levels was marginally significant between groups (F_4,18_ = 2.627; p = 0.06). Post hoc Dunnett’s test showed that serum MOG levels were significantly higher in rats administered 1 nmol/min of KYNA compared to PBS (p<0.05; Fig 5). Rats infused with 10 nmol/min of KYNA developed severe weakness and were terminated early, by day 5. Sufficient blood samples were not collected from these rats in relation to their poor clinical condition.

## Discussion

This is the first study documenting that the continuous long-lasting intrathecal infusion of KYNA in the spinal cord results in a specific damage to myelin and its loss with preservation of axons and oligodendrocytes and no cellular inflammatory infiltration. Extent of the myelin damage correlated with the increasing dose of applied KYNA. Moreover, the rats infused with the highest concentrations of KYNA (1 and 10 nmol/min) demonstrated adverse neurological signs including weakness and quadriplegia attributable to diffuse myelin damage. Although lower doses of KYNA (0.01 nmol/min and 0.1 nmol/min) produced significant loss of myelin noticeable neurological deficits were not discovered. An intrathecal infusion of KYNA at the dose 50.000-fold lower (0.0002 pmol/min), which can be considered “physiologically relevant” resulted in rare scattered swollen myelin sheaths.

The spatial arrangement of damage and loss of myelin, most severe in the sub-pial areas of the lateral and ventral columns and then gradually less severe to none in deeper areas suggests that the toxic effect of elevated concentration of KYNA is associated with its diffusion from the subdural space. This hypothesis is somewhat less applicable to the dorsal column where the centrally located fasciculus gracilis contained large numbers of damaged myelin sheaths or naked axons while the adjacent fasciculus cuneatus had remarkably fewer damaged myelin sheaths. The reason for this contrasting discrepancy is unknown at the present, it may be however suggestive of different susceptibility of oligodendrocytes in either axonal tract to the myelin-damaging action of KYNA.

The morphology of myelin damage induced by the infusion of KYNA was consistent in all affected areas of white matter. The damaged myelin sheaths had individual or stacks of lamellae split from each other in segmental or in diffuse fashion at the intraperiod line. The intraperiod line, the place where two external faces of the cytoplasmic membrane are apposed is enriched in proteolipid protein [28]. The major dense line enriched in myelin basic protein (MBP) was found to be intact even in individually splintered lamellae. This morphology may indicate a specific mechanism of myelin damage and eventually its loss related to a peculiar adverse effect of KYNA on oligodendrocytes and perhaps directed from KYNA-influenced oligodendrocytes. Myelin loss appeared to occur in an orderly fashion and the dehiscence of the lamellae at the level of the intraperiod line rather than MBP-enriched and potently antigenic major dense line. Although the oligodendrocytes in the areas of myelin damage and loss were preserved they appeared abnormal with small amount of cytoplasm, poor in organelles and with retracted processes.

It is noteworthy, that despite remarkable damage and loss of myelin a vast majority of axons remained morphologically intact and only scattered axons appeared dark, shrunken, surrounded by typically splintered myelin sheath in the affected areas of the white matter. It is known, that myelin potently inhibits axonal plasticity and regeneration in the adult CNS [29,30] and its removal from axons without considerable damage to glial cells and induction of inflammatory reaction has not been achieved with success previously but is desirable since naked axons preserve their plasticity in the adult age [31,32] and can regenerate after their transection [32,33]. It remains to be seen in studies allowing longer survival after the subdural infusion of KYNA whether naked axons regain their plasticity such as by sprouting [31–33] and whether persisting oligodendrocytes regain their ability to myelination the surrounding naked axons after the arrest of KYNA administration. On the other hand, naked axons in demyelinated models are known to influence proliferation of oligodendrocytes and stimulate their demyelinating efforts [33].

In an attempt to determine whether myelin damage is associated with elevation of myelin proteins in serum we measured MOG of rats infused with 0.1 nmol/min of KYNA. Although the numbers of the rats sampled was low, the levels of MOG appeared to increase in parallel with the dose of KYNA applied. MOG, a glycoprotein exclusively expressed in the white matter, is a minor myelin protein expressed on the, outermost surface of the myelin sheath and on the myelinated oligodendrocytes [34–40]. In animal models of experimental allergic encephalomyelitis (EAE) it has been identified as the target of demyelinating autoantibodies [34,35,41–45]. High titres of anti-MOG autoantibodies have been detected in paediatric patients with a variety of demyelinating inflammatory diseases but in the adult cases of multiple sclerosis the role of MOG is controversial since the specific antibody levels are not always elevated and the increase in titters is not robust [34,46–52].

Damage to myelin sheaths and outright loss of myelin evoked by intrathecal infusion of KYNA was not associated with cellular inflammatory infiltration as is commonly seen in demyelinating diseases such as the multiple sclerosis or spinal cord injury [31–33], where massively damaged myelin leads to severe infiltration by leukocytes [53]. Mechanism(s) of the apparently non-inflammatory removal of myelin in KYNA-treated spinal cord white matter is unknown at this point but lead us to call it myelin loss rather than demyelination, a term reserved to myelin loss associated with a severe inflammatory response.

The damage and loss of myelin in KYNA treated rat was associated with locally diffuse and severe astrogliosis. This reaction can be considered as a response of the CNS to the tissue damage. Interestingly, astrocytes are known as a main source of indigenous KYNA in the CNS [1,2]. KYNA is well known as the NMDA and α7nACh receptor antagonist. It is also known that excessive blockade of these receptors can alter brain function reducing the brain plasticity [54,55]. Therefore, the elevation of brain KYNA has been speculatively linked to neuropsychological disorders with impaired cognitive function [11,13,16,55]. It should be emphasized, that myelin abnormalities have been observed in several disorders with elevated KYNA content. Biopsy and post-mortem studies in patients with schizophrenia have documented loss of myelin sheath compactness, inclusion between lamellae sheaths and formation of concentric lamellar bodies [56]. Similarly, disarrangement of myelin structure has resulted in functional degradation of important neural circuits impairing cognitive and behavioural function [57]. Myelin disorders are also observed in multiple sclerosis and amyotrophic lateral sclerosis [58]. Although, the aetiology and mechanisms leading to myelin damage have not been determined, accumulating data presented a strong relationship between myelin disorders and NMDA receptors [27,59,60]. Since KYNA is an endogenous antagonist of NMDA receptors and the acute intrathecal administration of KYNA impaired motor function probably via blockade of NMDA receptors [61,62], and our results indicate that long-lasting intrathecal administration of KYNA produce myelin damage and elevation of serum MOG, it can be speculated that prolonged and excessive blockade of NMDA receptors may lead to myelin destruction. However this hypothesis required further, specific studies.

An intrathecal infusion of KYNA at the dose of 0.0002 pmol/min did not practically affect motor function of rats. This finding further confirms the concentration-dependent effect of KYNA. Based on the results of *in vitro* electrophysiological examinations, the idea that KYNA in the concentration range between a few hundred nanomolar and micromolar displays different effects has been previously presented [9]. In our study, infusion of KYNA in higher amount (1 nmol/min– 10 nmol/min) caused a dose-dependent behavioural dysfunctions. These rats presented moderate to severe imbalance and ataxia, and loss of body weight. Similar effects were described by Safrany-Fark and colleagues, who observed short-lasting hyperactivity after single dose of KYNA [22]. In another study, the intrathecal administration of KYNA as reported to cause a dose-dependent antinociception and long-lasting motor impairment [21]. Noteworthy, elevated levels of KYNA in CSF have been observed in relapsing-remitting multiple sclerosis patients, which is a demyelinating disease [63]. In the present study we observed myelin disorders following raised CSF KYNA concentration after continuous intrathecal infusion, and rats with severe myelin disorders presented mild to moderate hind end weakness. Based on this observations we can speculate that increase in CSF KYNA concentration may impair motor function via myelin injury and myelin loss.

In summary, we demonstrated that intrathecal infusion of KYNA caused injury and loss of myelin with preservation of axons and oligodendrocytes and development of astrogliosis but with no inflammatory response. The loss of myelin was associated with elevation of MOG in the blood serum. We suggest, that subdural infusion of high dose of KYNA can be used as an experimental tool for the study of mechanisms of myelin damage and regeneration. On the other hand, the administration of low, physiologically relevant doses of KYNA may help to verify whether KYNA plays role in the control of physiological myelination process.
